# Profile: Dr Simon Sinclair

**DOI:** 10.1192/pb.bp.114.049809

**Published:** 2014-12

**Authors:** Julia Bland

**Affiliations:** June 2014

Simon Sinclair’s dark beady eyes confront the onlooker like a spiky intelligent bird, looking for a worm to pounce on. Useful in the context of a participant-observer study of medical acculturation which he published as *Making Doctors* (1997). This laser beam of anthropological analysis was recently cited by the Royal College of Psychiatrists’ President Simon Wessely as the book all doctors should read.

Sinclair has now turned his attention to a crucial question for psychiatry: Why is the profession so unattractive to junior doctors? He answers this with an extension of his analysis of the kinds of knowledge and preoccupations that are silently absorbed in medical training, which he calls ‘dispositions’, attitudes which, while unspoken, determine the behaviour of medical students and the developing prejudice against psychiatry over the course of medical training. This is the hidden, unofficial curriculum at medical school, which inexorably leads to the devaluation of psychiatry. In a soon to be published paper, he details this unfortunate pattern and suggests logical remedies to tackle misunderstanding and prejudice head on, thereby enhancing recruitment, a major preoccupation of current and future presidents of the College. His own route to psychiatry illustrates aspects of his thesis, namely the way in which medical students are inducted into seeing themselves and their doctoring as problem-solving scientists, with an accompanying relegation of psychiatry as ‘unscientific’ and lacking in clarity.

Sinclair was born into an academic family; both his parents were senior civil servants and his maternal grandfather was Provost of Oriel College at Oxford University and a noted classical scholar. His grandson followed the family tradition, excelled at Latin and Greek, but changed to science in order to do medicine. At 16, he had an inkling that psychiatry might lead to some ‘vicarious understanding of myself through the medium of other people’. He wanted to know (an unusual entree into psychiatry), ‘why Dido and Aeneas had so failed to communicate’, with Aeneas abandoning her and her subsequent suicide. This curiosity then got lost for 15 years as he threw himself into the project of rationality and science which, while intellectually intriguing, felt like a limiting blind alley which left him medically qualified, but furious and disappointed.

The scientific education of medicine he found excluding and alienating from contemporaries: ‘I felt a side of me was atrophying’. There was one memorable moment of comfort when, in the psychology library, he found a picture of ‘a little African girl turning her eyes down to look at a fly on the end of her nose’. This was a rare glimpse of a person rather than a psychological abstraction, even among psychologists, whose intellectual preoccupations in Oxford at that time were ‘statistics, perception, the physical attributes of the eye . . . interesting enough but not pertinent to my questions’.

‘We medical students were taken to the Warneford (psychiatric) hospital once, to hear a patient recount her experience of mania, under the ”abnormal psychology” heading, but no links were made between the science and the clinical world’.

After this dismal experience of academic psychology at Oxford, he tried clinical medicine at Guy’s and St Thomas’ Hospital. This was not an improvement. Guy’s was ‘the most toxic institution I’ve ever been involved with . . . careless. There was a failure to provide any context other than its own from which to see things’. He acknowledges ruefully that he has always had a ‘deeply ambivalent relationship with institutions. I know them very well, these ”greedy institutions”, their blind alleys, their inability to know what they are up to’.

After house jobs and a job in accident and emergency medicine, he headed to the hills of Nepal, enjoyable years tramping about with a dog, delivering drugs and practical medical care to remote villages with the British Nepal Medical Trust. Problems were simpler and more severe than in the UK, more amenable to rational intervention. It was a chance ‘to get a different kind of grip, not just to learn things as facts. Also I was learning language and custom . . .

**Figure:**
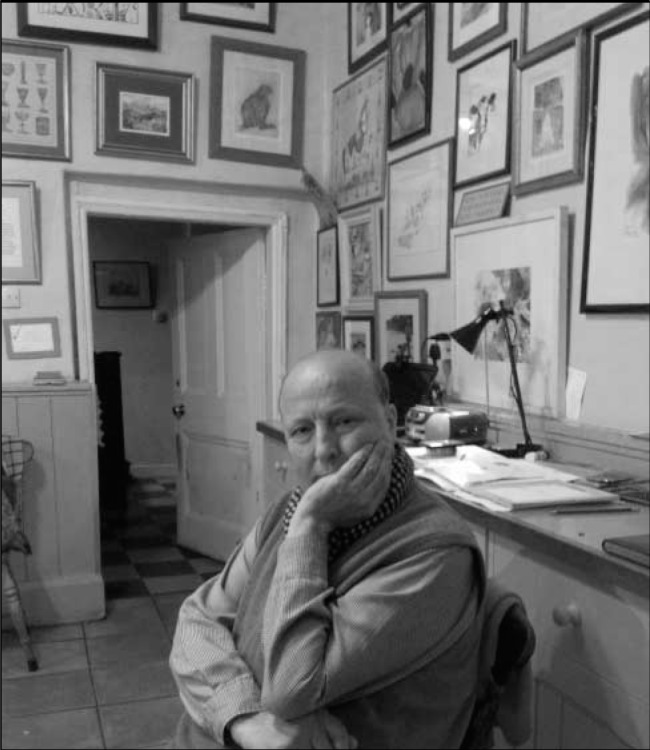

so different to medical school’. He returned to the UK after 3 years, with a medical student wife and baby. He considered a career in public health in low-income countries and did the Diploma in Tropical Medicine and Hygiene. But psychiatric locums seemed practical and he began these in 1984 in and around London.

Psychiatry was surprisingly congenial: ‘listening to people, trying to convert it into a medical phrase’. Sinclair decided to apply for a training rotation. He was repeatedly interviewed and then rejected, ‘because I told the truth’. Example: Interviewer – ‘Why do you want to do psychiatry?’, Sinclair – ‘I find it intriguing’ (one of his favourite words); Interviewer – ‘Where do you see yourself in 5 years’ time?’, Sinclair – ‘I can’t tell the future, I might be run over by a bus’; Interviewer – ‘How do you see the field developing?’, Sinclair – ‘That’s what I’m here to learn’. Unsurprisingly, this smartass honesty went down badly and he was advised that he was ‘not quite ready’. The Sinclair family intellectual arrogance is not confined to Simon. His brother, when asked how his PhD *viva* had gone, replied ‘They asked me a whole lot of impertinent questions’.

After some badly-needed advice on how to be interviewed, he was accepted to the Oxford rotation but remained an awkward customer, not an insider. In one job he found himself attending the inquest of a woman who had died subsequent to excessive haloperidol. The consultant who had prescribed the drug did not appear in court or give evidence, but Sinclair did. He realised then that there was ‘no such thing as consultant responsibility in law’.

While remembering with horror the casual way in which high-dose antipsychotics were prescribed in those days, he is nostalgic for the use of the ward as a therapeutic refuge. He cites the example of an elderly homeless man with schizophrenia who presented at the beginning of winter and the consultant’s response was to ‘have him in for a few months’. Sinclair recognises how his own attitude has evolved and how patients’ symptoms were not then seen in the context of their families. Although the much-cited work on expressed emotion by Leff *et al* was well known, there was no effect on practice or thinking (and it can easily be argued there is still far too little of it now). Professor Keith Hawton stood out as a teacher in some ways: all new patients had to be videotaped and the tape played at the ward round. There was a real attempt to ‘work out what was going on and write it down’. The ward was the focus for in-patients, out-patients and day-patients, so there was some possibility of continuity of care and Sinclair saw the centrality of the nurses’ contribution to this.

Having passed the Royal College of Psychiatrists’ membership examination, Sinclair felt liberated to ‘do it my way . . . listening to the patient properly, not asking questions in the order that you’re taught, or in the words you’re meant to use’. Then the big break into anthropology came. Sinclair was working part-time on the ‘married women’s scheme’, looking after his daughter with Down’s syndrome, and decided to do a part-time anthropology degree at the London School of Economics, encouraged by the well-known general practitioner and epidemiologist Muir Gray (who happened to be working in the Oxford deanery at the time). With huge relief, at the London School of Economics Sinclair reconnected to his curious 16-year-old self. He was in a discipline where ‘words didn’t just mean things. I was back with the little African girl with the fly on her nose, with other people’s real lives. Looking at the different ways in which power is exercised, the nature of ritual performance’. He noticed that anthropology uses the anatomical method of looking at structure and function applied to societies. He was having such fun he decided to do a PhD. Domestic commitments made working abroad impossible, so he turned to the socialisation of doctors. He wanted to understand how his own original intention to do psychiatry had been ‘wiped from my mind’ by the medical training.

With a conviction of the centrality of anatomical dissection as an experience distinguishing doctors, he got himself back into the dissecting room at University College London. He studied the language, ideas and experiences of medical students at different stages of training and house jobs. He was interested in the ‘backstage stuff’, the language in the bar, the crude jokes, the overt sexism. The book *Making Doctors* (recommended by our President Simon Wessely to all doctors) describes these ‘dispositions’, this other way of knowing than factual knowledge.

These dispositions or ‘cognitive categories’ are knowledge, responsibility, status and economics, with psychiatry scoring very low on status (‘not proper doctors’), high on responsibility (‘scary’) and low on knowledge (‘not proper science’). Contrast this with medicine (high on knowledge) or surgery (high on responsibility). Dermatology was seen as good economically (‘soft option, easy hours’), but low on responsibility and very low on status. Sinclair illustrates how these unspoken hierarchical attitudes are promulgated to medical students throughout training. Within the undergraduate curriculum, psychology and sociology are low-status subjects, criticised as woolly, unscientific. He recorded students’ disparaging comments, for example ‘I waited for half an hour (in a sociology lecture) for a fact to write down’ (p. 166) or similarly with psychology: ‘It’s all a matter of opinion’.

Sinclair points out how psychiatry lacks concrete evidence of pathology (in spite of the mental state examination itself being modelled on the physical examination) and that much treatment is ‘only talking’ and responsibility is shared among a multidisciplinary group. In addition, the patients are often ‘uncooperative’, i.e. unattractive and a nuisance to the junior in a hurry. Often self-inflicted injury is seen as super uncooperative: the registrar’s comment on an patient who had taken an overdose who discharged himself against medical advice: ‘good riddance to bad rubbish’ (p. 295).

Having got his doctorate at the London School of Economics and his Certificate of Completion of Training in psychiatry, he was tempted 15 years ago by a consultant job in Durham, which offered the opportunity to teach medical students in the new medical school. The idea was to help students learn using non-medical experiences such as working with the Riding for the Disabled charity, helping older people, and teaching in a style informed by sociology and anthropology. This promise did not materialise, as his time for writing and teaching was gradually eroded by clinical demand. He built up a successful community team over 10 years, only to see it disbanded: ‘heartbreaking’. In a culture which increasingly demands that schizophrenia be ‘treated’ within 2 years, he argued that ‘we have patients that need long-term support and I know them’, but a nurse consultant responded sarcastically ‘Gosh, Dr Sinclair, you must be a really good doctor if you can keep people out of hospital seeing them every 3 months’. Of course, if proper value is given to an ongoing therapeutic relationship that is precisely what a good consultant can do.

His last years at work have been on in-patient wards, where ‘disasters are taken more seriously’, but now his own ill health has obliged him to retire (not early). His commitment to his patients was touchingly referred to in one of his leaving cards: ‘the best consultant I ever worked with in my 20 years as a psychiatric nurse’. Now he sees the local community mental health teams ‘going to pot. There is so much paperwork the nurses just buckle’, but he is encouraged by the new longer GP training including psychiatry and (eccentric himself) he urges a society more tolerant of oddity and the avoidance of premature mental illness labelling.

Now for his advice on recruitment into psychiatry. He and his co-author, Dr Padma Suresh Babu, analysed (grounded theory) the contents of groups conducted with fifth-year medical students. They found comments on the dubious credibility of psychiatry as a medical specialty on the basis of its complexity and uncertainty. Psychiatric illness certainly lacks the clarity of a broken leg. The student experience of psychiatric patients was uneasy; they were ‘difficult’, meaning ‘uncooperative’, there was a lack of demonstrable physical signs and the problems were ‘not resolved’. Analysed under Sinclair’s medical ‘dispositions’, the study led to interesting conclusions.

Knowledge: psychiatry was seen as ‘very theoretical’ and the students had ‘difficulty with concepts’. Responsibility: the students objected to the inconclusiveness of the interventions – ‘it’s just talking and writing’, ‘not like hands on actual stuff’. Economy: the students complained of poor return on time allocated – ‘you’ve got an hour to get the information out, and you could be completely wrong anyway’. They also worried about the potential waste of their hands on intervention training, becoming deskilled. Cooperation: psychiatric patients were found to be perceived by the medical students as scary and intimidating. Idealism: the students displayed some respect for psychiatrists – ‘you’ve got to be strong . . . interested in people’. Status: this was inevitably low given the scores of psychiatry within the cultural categories above and indeed, these groups did produce the old clichés of ‘psychiatry is not real medicine’ and ‘all psychiatrists are crazy’.

Sinclair also identifies some external factors contributing to the low status of psychiatry within medical schools. These include the relatively small amount of time in the crowded syllabus allowed for psychiatric teaching, the academic/clinical split in psychiatry, leading to clinical teaching sometimes being delivered by busy consultants who are not interested in teaching, and the selection of ‘convergent’ rather than ‘divergent’ thinkers into medical school, as demonstrated by chemistry being more crucial than biology at A-levels.

Of course, this is in the overall context of stigma in relation to mental illness and psychiatrists, both within and outside medicine. Sinclair & Babu quote Light (1980): ‘Psychiatry rests its claim for professional status on a profession that is hostile to it’. The remedy which Sinclair proposes is about including his insights into student attitudes and taking them on directly. He recommends stressing the similarities with other medical specialties and explaining the differences.

Under ‘knowledge’, this would mean that while accepting the relative complexity and inaccessibility of the brain compared with the heart (just a pump, after all), the advances in neuroscience need to be emphasised. Also, research difficulties and successes need to be explained, as well as the evidence-based approach. Under experience, Sinclair urges the teachers to explain the difference between taking the history and taking the mental state examination, as well as the equivalence between physical signs and psychopathological findings. Under ‘responsibility’, stress the crucial engagement process, the chronicity of many psychiatric illnesses (like diabetes, arthritis etc.), the legal/ethical subtlety of the job under the Mental Health Act and the effectiveness of evidence-based treatments. Under ‘cooperation’, stress that it is unusual compared with other medical disciplines to spend an hour alone with the patient and that can be alarming initially; that the patient’s illness affects their response as someone in physical pain may also be effected; and that there are particular skills in interviewing, including transcultural sensitivity. Under ‘economy’, point out how subsequent contact with a patient is enhanced by spending the time on the initial interview and consider encouraging students to follow particular patients over time rather than seeing a series. Under psychiatry as a waste of medical training, it can be argued that physicians do not do surgery or *vice versa*, and that medical training is vital given extensive and under-recognised comorbidity of physical and mental ill health, with the expansion of liaison psychiatry. Under ‘idealism’, the suffering of mental illness and the effectiveness of decent treatment can be emphasised, as well as the opportunity to use their intelligence to consider the major theoretical problems of psychiatry, the mind/brain divide, etc. Last, under ‘status’, we need to be more self-promoting, stressing the medical aspects of the job and the diversity of the specialty.

Since our president recently described recruitment into psychiatry as ‘falling off a cliff’, there can never have been a more pertinent moment for Simon Sinclair’s call to arms.

Simon Sinclair died of cancer on 4 September 2014. He leaves behind a devoted family and a wide group of friends and colleagues.

